# Bleeding patients on extracorporeal membrane oxygenation have reduced platelet aggregation and plasma fibrinogen: a longitudinal observational study

**DOI:** 10.1038/s41598-023-41773-3

**Published:** 2023-09-04

**Authors:** Christine Lodberg Hvas, Steffen Christensen, Camilla Mains Balle, Heidi Munk-Andersen, Anni Nørgaard Jeppesen, Anne-Mette Hvas

**Affiliations:** 1 Department of Anaesthesiology and Intensive Care Medicine, Aarhus University Hospital, Aarhus, Denmark; 2 Department of Clinical Medicine, Aarhus University, Aarhus, Denmark; 3 Department of Endocrinology and Internal Medicine, Aarhus University Hospital, Aarhus, Denmark; 4 Department of Cardiothoracic- and Vascular Surgery, Anaesthesia Section, Aarhus University Hospital, Aarhus, Denmark; 5 Faculty of Health, Aarhus University, Aarhus, Denmark

**Keywords:** Biomarkers, Cardiology, Platelets

## Abstract

This study investigated changes in coagulation and associations with occurrence of bleeding and thrombosis during extracorporeal membrane oxygenation (ECMO) therapy. The study included 100 adult ECMO-patients. Standard coagulation parameters, platelet aggregation and thromboelastometry (ROTEM^®^) were compared with healthy controls. Data on bleeding and thrombosis were collected until recovery or death. Mortality data were collected 30 days after weaning from ECMO. During ECMO therapy, 53 patients experienced at least one moderate or major bleed. Among these, 42 (79%) patients experienced the first bleeding on day 1 or 2. Platelet aggregation and ROTEM^®^ revealed a hypocoagulable state in ECMO patients when compared with healthy controls. Patients bleeding on day 1 or 2, had lower platelet count (p = 0.04), poorer platelet aggregation and lower levels of fibrinogen (p < 0.01) than patients not bleeding on day 1 or 2. Further, ROTEM^®^ clot propagation was reduced in bleeding patients (p < 0.001). Mortality was higher among bleeding patients than patients not bleeding on day 1 or 2 (67% versus 34%, p < 0.01). Congruity existed between ROTEM^®^ measurements and standard coagulation assays, but plasma fibrinogen had a stronger association with bleeding than ROTEM^®^ measurements. The present study does not support ROTEM^®^ analysis as a routine part of coagulation monitoring during ECMO therapy.

## Introduction

Use of extracorporeal membrane oxygenation (ECMO) has increased within the last decade, particularly the use of ECMO for extracorporeal cardiopulmonary resuscitation (ECPR) in patients with refractory cardiac arrest^1–4^. Mortality and frequency of bleeding and thrombotic complications vary between ECMO configurations, but bleeding has consistently been associated with mortality^5, 6^. Over the past years, the incidence of adverse events in general has decreased, but the incidence of medical bleeding remains unchanged^5^. Non-modifiable risk factors for bleeding or thrombosis, such as age, sex, and weight, have been identified using the Extracorporeal Life Support Organization (ELSO) registry^5, 6^, but very limited data exist on the direct impact of coagulation status.

The dynamic process of coagulation from activation of platelets and clotting factors to fibrinolysis can be exhaustively examined only by using a combination of dynamic assays, such as platelet aggregometry and global whole blood coagulation assays (e.g., thromboelastography [TEG] or rotational thromboelastometry [ROTEM^®^]), which are widely implemented in the handling of hemorrhage, especially during liver and cardiac surgery^7–9^.

Few studies have evaluated the use of TEG/ROTEM^®^ during ECMO therapy and small sample sizes hamper clear interpretation of the associations between changes in coagulation and the development of bleeding, thrombotic complications and mortality^10–16^.

Evaluation of platelet aggregation during ECMO therapy has been performed in few studies^16–18^. But as with TEG/ROTEM^®^, association between platelet aggregation and bleeding or thrombosis during ECMO therapy remains to be fully elucidated^17^.

The present study aimed to (1) investigate platelet aggregation, ROTEM^®^ and in vivo thrombin generation during ECMO therapy, (2) determine the prevalence of bleeding and thrombotic events during ECMO therapy and (3) investigate associations between coagulation parameters and bleeding or thrombotic events.

## Methods

The present study is a longitudinal single center cohort study conducted at the intensive care unit (ICU) at Aarhus University Hospital, Denmark, a tertiary ECMO referral center treating approximately 60 ECMO patients annually. Patients were included from May 2017 until December 2019. The project was approved by the local institutional board and the Danish Data Protection Agency (Ref. no. 1-16-02-712-17). According to the Danish Health Care Act, requirement for written informed consent was waived after formal review by the Health Ethics Committee of Central Region, Denmark. The study followed the 1964 Declaration of Helsinki and The Danish Data Protection Agency approved the study.

### Study population

Inclusion criterion was patients aged 18 years or older presenting with severe cardiac and/or pulmonary failure requiring ECMO therapy. Exclusion criteria were ECMO therapy following major cardiac surgery (post-cardiotomy VA-ECMO) as the use of heart–lung machine and concomitant high risk of post-operative bleeding influence coagulation. Further, patients were excluded if blood sampling for ROTEM^®^ and platelet aggregation analyses for logistical reasons were not possible latest on day 3 of ECMO therapy.

### ECMO management

All patients were treated at the discretion of the ICU/ECMO team and treatment followed in-house standards closely adapted to ELSO recommendations. ROTEM^®^ and platelet aggregation results from study blood samples were not available to the attending physician. Patients were cannulated with 17–23 Fr venous cannulas, and 15–21 Fr arterial cannulas, all jugular-femoral (veno-venous (VV)-ECMO) or femoral-femoral (veno-arterial (VA)-ECMO) cannulation. By standard, the ECMO circuit was inspected for fibrin and clot formation by experienced ECMO specialists every 8 h.

#### Anticoagulation

Patients received unfractionated heparin (UFH) as an intravenous bolus injection of 5000 IU upon ECMO cannula insertion. Heparinization was monitored by measurement of activated partial thromboplastin time (APTT, reference range 20–29 s) at least four times daily; the aim was 40–55 s in VV-ECMO patients and 50–65 s in VA-ECMO patients, obtained by continuous intravenous UFH. Antithrombin levels were kept above 80%. Platelet inhibitors were prescribed at the discretion of the attending cardiologist in collaboration with the ECMO specialist physician.

#### Transfusions and treatment of bleeding

Decision on platelet transfusions aimed to maintain platelet count above 50 × 10^9^/L. In case of bleeding complications, the transfusion threshold was increased to platelet count of 80–100 × 10^9^/L. The standard threshold for transfusion of red blood cells was a hemoglobin of 6.0 mmol/L (corresponding to 96.7 g/L).

In case of bleeding potentially leading to hypovolemia and subsequent limitations in running sufficient ECMO blood flow, a balanced transfusion strategy was applied with a 1:1:1 ratio of red blood cells, fresh frozen plasma, and platelets. This was supported by 24-h available ROTEM^®^ guided corrections, adding extra fibrinogen concentrate or fresh frozen plasma if needed. In case of fluid overload, prothrombin complex concentrate was considered and in case of refractory bleeding recombinant factor VII was available. In general, treatment of bleeding event was at the discretion of the attending physicians with special knowledge on ECMO therapy with support from a team of coagulation experts from the Department of Clinical Biochemistry.

### Clinical data

Clinical variables were extracted systematically from the patient medical records and ICU observation charts. These included: ECMO configuration; indication for ECMO therapy; comorbidities selected in accordance with the Charlson Comorbidity Index^19^; Sequential Organ Failure Assessment (SOFA) Score modified to use the Richmond Agitation-Sedation Scale^20^; daily evaluation of bleeding or thrombosis events until termination of ECMO support. Bleeding complications were classified according to the Global Utilization of Streptokinase and tPa for Occluded arteries (GUSTO) bleeding criteria^21^ and anatomical location. Occurrence of thrombosis was classified as arterial or venous. Further, 30-day mortality after ECMO weaning, transfusion of blood products, and daily therapy with pro- or anticoagulant medications were registered.

### Blood sampling and laboratory analyses

Blood for standard coagulation parameters were collected within the daily routine rounds. The first blood sample for platelet aggregation, ROTEM^®^ analysis or in vivo thrombin generation was collected on the first possible weekday following ECMO initiation, latest on day 3. Blood samples were then collected on weekdays until day 7. Thereafter blood samples were collected on days 14 ± 2 days and 21 ± 2 days, if the patient was still receiving ECMO therapy. Blood was drawn from a non-heparinized arterial cannula already in place.

#### Platelet aggregation

Platelet aggregation was investigated using the Multiplate^®^ R Analyzer (Roche, Basel, Schwitzerland) using whole blood collected in hirudin tubes^18^. Adenosine diphosphate (6.5 μM, ADPtest), thrombin-receptor-agonist-peptide-6 (32 μM, TRAPtest), and arachidonic acid (AA) (0.5 mM, ASPItest) (all from Roche, Basel, Switzerland) were used as agonists. Platelet aggregation results are reported as area under the curve (AU × min).

#### Thromboelastometry, ROTEM^®^

Blood for ROTEM^®^ (Instrumentation Laboratory, Bedford, MA) was collected in citrated tubes. Standard protocols for EXTEM, INTEM, FIBTEM, and HEPTEM were performed. The following parameters were registered: clotting time (CT, s), time to maximum velocity (t-MaxVel, s), maximum velocity (MaxVel, mm/s) and maximum clot firmness (MCF, mm). Calculation of the platelet component, maximum clot elasticity (platelet MCE), was derived from ROTEM^®^ tests as described by Solomon et al.^22^. First, MCE was calculated for both EXTEM MCF and FIBTEM MCF using the following formula: MCE = (100 × MCF)/(100 − MCF) for each parameter. Second, clot elasticity attributable to platelets was calculated as: platelet MCE = MCE EXTEM − MCE FIBTEM.

#### In vivo thrombin generation

In vivo thrombin generation was determined by thrombin–antithrombin (TAT) complex plasma concentrations. Blood was collected in citrated tubes and analysed with commercial enzyme-linked immunosorbent assay (ELISA) kit (Enzygnost^®^TATmicro, Siemens, Marburg, Germany).

#### Healthy controls

For Multiplate^®^ and ROTEM^®^, 80 healthy individuals served as controls (All healthcare workers. For Multiplate^®^, two are missing), presented in a previous publication^23^. For TAT measurements, 122 healthy blood donors served as healthy controls and established a reference interval, also previously published^24^.

#### Markers of organ function and standard coagulation assays

Platelet count, international normalized ratio (INR), APTT, antithrombin, fibrinogen, D-dimer, hemoglobin, and markers of inflammation and organ function were analyzed at the automated routine laboratory according to ISO:15189 accredited protocol.

### Data management and statistics

Study data were managed using REDCap electronic data capture tools^25^. Statistical analysis and graphs were performed using GraphPad, Prism 9 (GraphPad Software Inc., CA, USA). All data are presented as median with interquartile range (IQR) for uniformity, as most variables did not follow normal distribution. In figures, data are presented in Tukey plots. To minimize missing data, blood samples were grouped into the following days: day 1 (± 1), day 4 (± 1), day 7 (± 1) and day 14 (± 2). Samples from day 21 (± 2) are not presented as only four patients were still on ECMO for this long. Changes over time are presented graphically, but not tested statistically due to a significant drop-out because of weaning or death. Differences between ECMO patients and healthy controls and between patients with and without bleeding were assessed with unpaired *t*-tests or with Mann–Whitney test, when appropriate. Distribution of categorical variables between groups was assessed with Fisher’s exact test or Chi-square test when appropriate. All tests of significance were two-tailed; p < 0.05 was considered significant.

## Results

A total of 100 patients were included for analysis while 24 patients were excluded as they were transferred either from cardiac surgery (post-cardiotomy VA-ECMO) or from other hospitals later than day 3 of their ECMO therapy.

Patient demographics, clinical characteristics, details on ECMO therapy, antiplatelet therapy and mortality are presented in Table 1. In total, 31% of patients received VV-ECMO and 69% VA-ECMO. The main indication for VA-ECMO was ECPR, initiated in 39% of all patients. A total of 778 days of ECMO was evaluated with median duration of 6 (3–10) days.

**Table 1:** Demographic and clinical data on 100 patients receiving extracorporeal membrane oxygenation therapy. Data are provided as median [interquartile range] or n (%) as appropriate.

	Value
Demographics	
Age, years	56 [45–64], range 19–83
Sex, male/female	67/33
BMI, kg/m^2^	28 [25–32]
ECMO details	
VV-ECMO	31 (31%)
VA-ECMO	69 (69%)
VA-ECMO + Impella	6 (6%)
Change of ECMO mode, from VA to VV or combined VA/V	7 (7%)
ECMO indication	
Pulmonary	35 (35%)
Cardiac	26 (26%)
ECPR	39 (39%)
Duration of ECMO, days	6 [3–10], range 1–52
1–3 days	36
4–7 days	31
8–13 days	16
≥ 14 days	17
Reason for termination of ECMO	
Recovery	67 (67%)
Mortality	33 (33%)
Dialysis during ECMO treatment	58 (58%)
Comorbidity and SOFA score	
Charlson’s Comorbidity Index	0 [0–1], range 0–4
No comorbidities	55 (55%)
Acute myocardial infarction	8 (8%)
Chronic heart failure	13 (13%)
Chronic pulmonary disease	8 (8%)
Diabetes	17 (17%)
SOFA score, day 1	13 [11–15], range 6–22
Antiplatelet therapy	
Day 1, single/dual	10 (10%)/9 (9%)
Day 4, single/dual	3 (5%)/9 (14%)
Day 7, single/dual	1 (3%)/2 (5%)
Day 14, single/dual	0 (0%)/0 (0%)
Mortality	
Mortality at ECMO weaning (of total n = 100)	33 (33%)
30-day mortality after successful weaning (of total n = 67)	15 (22%)
Alive 30 days after weaning (of total n = 100)	52 (52%)
VV mortality, on ECMO or within 30 days after weaning (of total n = 31)	10 (32%)
VA (not ECPR) mortality, on ECMO or within 30 days after weaning, (of total n = 30)	12 (40%)
ECPR mortality, on ECMO or within 30 days after weaning, (of total n = 39)	26 (67%)

*IQR* interquartile range, *n* number, *ECMO* extracorporeal membrane oxygenation, *VV* venous-venous, *VA* venous-arterial, *ECPR* extracorporeal cardiopulmonary resuscitation, *SOFA* sequential organ failure assessment score.

Level of comorbidity was low according to Charlson’s Comorbidity Index and half of the patients were registered as having no comorbidities. Disease severity was stable during ECMO therapy as median SOFA score day 1, 4, 7 and 14 ranged between 12 and 15 (IQRs 9–18).

Markers of organ function and conventional markers of coagulation (INR, APPT, platelet count, antithrombin, fibrinogen, D-dimer) are presented in Table 2. Further, APTT and infusion rate of UFH are depicted. Median and IQR of APTT was within or below target range except for the 75th percentile for VA-ECMO day 1. Over time, infusion rates of UFH doubled while APTT remained stable.

**Table 2:** Biochemical parameters and infusion rate of unfractionated heparin day 1, 4, 7 and 14 for 100 patients during extracorporeal membrane oxygenation therapy. All median (IQR).

	Day 1 n = 100	Day 4 n = 63	Day 7 n = 38	Day 14 n = 13
Organ markers (reference interval)				
Hemoglobin (7.3–10.5 mmol/L)	6.5 (5.9–7.1)	6.2 (5.9–6.6)	6.1 (5.9–6.3)	6.1 (6.0–6.5)
Haptoglobin (0.35–2.05 g/L)	0.53 (0.15–1.6)	0.54 (0.06–1.19)	0.33 (0.06–1.19)	0.09 (0.06–0.87)
LDH (105–255 U/L)	806 (413–1391)	531 (435–914)	461 (379–743)	475 (381–961)
Leukocytes (3.5–10.0 × 10^9^/L)	11.9 (7.9–17.6)	12.9 (8.7–18.5)	18.3 (13.3–22.4)	14.5 (8.3–21.5)
CRP (< 8.0 mg/L)	88 (37–193)	186 (114–242)	151 (52–196)	83 (39–248)
Creatinine (45–105 µmol/L)	150 (102–224)	121 (78–174)	133 (81–181)	115 (54–173)
Carbamide (2.6–8.1 mmol/L)	10.4 (7.6–14.2)	11.7 (8.2–15.1)	15.2 (11.5–20.6)	12.4 (9.6–21.4)
Albumin (36–45 g/L)	33 (29–38)	31 (26–38)	30 (24–36)	28 (25–35)
Bilirubin (5–25 µmol/L)	23 (15–39)	35 (17–63)	35 (16–63)	25 (17–48)
ALAT (10–70 U/L)	157 (47–390)	75 (45–156)	60 (40–103)	55 (28–86)
Lactate (0.5–2.5 mmol/L)	2.9 (1.7–5.9)	1.7 (1.2–2.5)	1.6 (1.3–2)	1.4 (1.3–1.7)
pH (7.37–7.45)	7.38 (7.33–7.42)	7.43 (7.39–7.46)	7.40 (7.37–7.47)	7.39 (7.37–7.44)
Coagulation parameters				
Platelet count (145–400 × 10^9^/L)	141 (91–202)	73 (46–141)	110 (74–199)	168 (112–220)
INR (< 1.2)	1.5 (1.3–1.8)	1.3 (1.1–1.4)	1.2 (1.0–1.3)	1.1 (1.0–1.2)
Antithrombin (80–120%)	66 (54–82)	86 (71–100)	96 (82–118)	90 (85–101)
Fibrinogen (187–408 mg/dL)	279 (190–415)	524 (412–663)	578 (401–728)	493 (306–762)
D-dimer (< 0.80 mg/L FEU)	8.8 (3.4–18)	5.1 (2.2–10.9)	8.3 (3.9–19.1)	11.3 (1.9–20)
APTT, all patients (20–29 s)	42 (33–60)	47 (42–51)	44 (39–53)	43 (34–51)

	Day 1	Day 4	Day 7	Day 14
APTT, seconds, by ECMO type (target)				
VV, n = 33 (40–55 s)	37 (30–47) n = 30	43 (39–49) n = 27	42(38–46) n = 24	42 (32–46) n = 7
VA (not ECPR), n = 30 (50–65 s)	47 (35–68) n = 28	46 (44–55) n = 18	45 (37–56) n = 8	49 (45–56) n = 4
ECPR, n = 39 (50–65 s)	49 (37–65) n = 39	50 (46–53) n = 17	57 (53–64) n = 6	38 n = 1
UFH, IE/h, all patients	500 (500–800)	875 (600–1200)	1100 (800–1500)	1350 (1000–1800)
VV ECMO	700 (500–800)	1000 (650–1400)	1200 (800–1500)	1500 (1150–1850)
VA ECMO (not ECPR)	500 (500–800)	800 (500–1000)	900 (738–1525)	1225 (1025–1688)
ECPR	500 (500–800)	700 (563–1175)	950 (775–1275)	700

Coagulation parameters, day 1, by ECMO type	VV ECMO	VA ECMO (not ECPR)	ECPR	
Platelet count (145–400 × 10^9^/L)	119 (88–208)	119 (72–163)	153 (129–194)	
INR (< 1.2)	1.4 (1.1–2.1)	1.6 (1.4–2.2)	1.4 (1.3–1.6)	
Antithrombin (80–120%)	79 (65–90)	64 (50–80)	66 (56–75)	
Fibrinogen (187–408 mg/dL)	422 (293–656)	247 (192–338)	231 (184–310)	
D-dimer (< 0.80 mg/L FEU)	3.9 (3.2–13.6)	6.7 (2.2–10.8)	13.6 (6.3–20)	

Reference ranges for hemoglobin, creatinine, carbamide, ALAT and platelet count are combined for men and women, and for haptoglobin, LDH, carbamide, albumin and fibrin D-dimer across ages.

*IQR* interquartile range, *LDH* lactate dehydrogenase, *CRP* C-reactive protein, *ALAT* alanine transaminase, *INR* international normalized ratio, *APTT* activate thromboplastin time, *FEU* fibrinogen equivalent unit, *ECMO* extracorporeal membrane oxygenation, *VV* venous-venous, *VA* venous-arterial, *ECPR* extracorporeal cardiopulmonary resuscitation, *UFH* unfractionated heparin.

### Platelet aggregation

**Figure 1:**
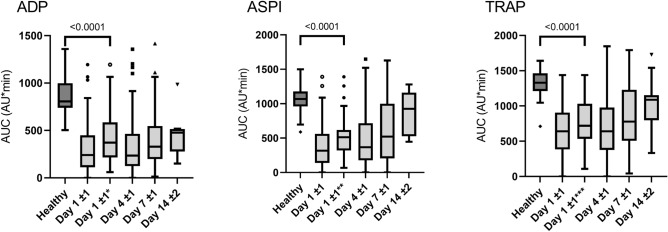
Measurements of platelet aggregation with the agonists adenosine diphosphate (ADPtest), arachidonic acid (ASPItest) and thrombin-receptor-agonist-peptide-6 (TRAPtets) from 100 patients on days 1 ± 1 (n = 87), 4 ± 1 (n = 74), 7 ± 1 (n = 44), and 14 ± 2 (n = 11) after initiation of extracorporeal membrane oxygenation therapy. Day 1* excluding patients treated with ADP inhibitor (n = 13), with platelet count < 100 × 10^9^/L (n = 25) or both (n = 4). Day 1** excluding patients treated with acetylsalicylic acid (n = 14), with platelet count < 100 × 10^9^/L (n = 23) or both (n = 6). Day 1*** excluding patients with platelet count < 100 × 10^9^/L (n = 29).

Measurements of platelet aggregation are presented in Fig. 1. On day 1, platelet aggregation was reduced for all three agonists when compared with healthy controls (all p < 0.0001). The reduction remained significant (all p < 0.0001) when excluding patients, who had a platelet count below 100 × 10^9^/L or who had received either acetylic salicylic acid (ASA) from the ASPItest or ADP inhibitors from the ADPtest.

Over time, median platelet aggregation increased in the ASPItest, possibly due to a decreased use of ASA (Table 1), while the use of ADP inhibitors remained stable as so did platelet aggregation measured by ADPtest. However, platelet aggregation measured by all three agonists remained below the level of the healthy controls (Fig. 1).

### ROTEM^®^ and standard coagulation markers

**Figure 2:**
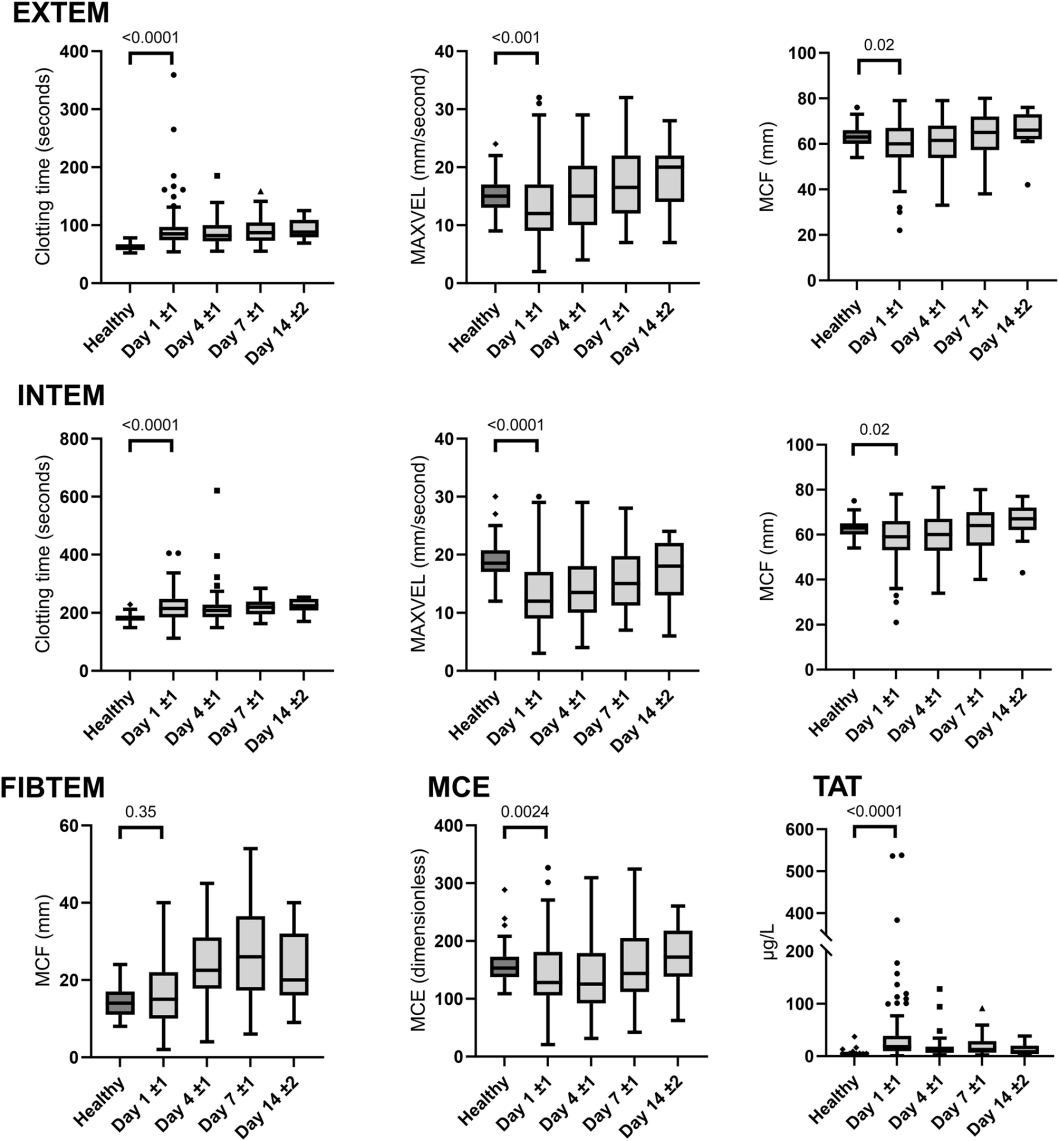
Thromboelastometry (ROTEM®) results and thrombin–antithrombin (TAT) complex plasma levels from 100 patients on day 1 ± 1 (n = 87), 4 ± 1 (n = 74), 7 ± 1 (n = 44), and 14 ± 2 (n = 11) during extracorporeal membrane oxygenation therapy. Selected results from EXTEM, INTEM and FIBTEM assays are presented, as well as the calculated platelet maximum clot elasticity (MCE). P-values reflect comparison to a healthy control group. *MAXVEL* maximum velocity, *MCF* maximum clot firmness, *MCE* maximum clot elasticity, *TAT* thrombin–antithrombin complex.

Results from ROTEM^®^ analysis are depicted in Fig. 2. On day 1, ROTEM^®^ revealed a hypocoagulable profile in ECMO patients in comparison with healthy controls. Clot initiation was prolonged in accordance with the slightly prolonged INR and the prolongation of APTT attributable to the infusion of UFH. The correlation between the 326 pairs of APTT and INTEM CT measurements was significant and moderate, Spearman r_s_ 0.53 (95% CI 0.44–0.60, p < 0.0001). Clot propagation was slower than in healthy controls, while FIBTEM clot firmness was preserved. Median fibrinogen level was within reference range and hence in accordance with the maintained FIBTEM MCF. Platelet MCE was significantly lower in ECMO patients than in healthy controls.

### In vivo thrombin generation

In vivo thrombin generation was evaluated by measurement of TAT complex levels as shown in Fig. 2. TAT was highest on day 1 with 65% of the patients having levels above our in-house established reference range (≤ 13 µg/L).

### Bleeding complications

In total, 66 patients suffered at least one bleeding event during ECMO therapy. The primary sites of bleeding were from the cannulation sites and from skin or mucosa, found in 52 (79%) patients. Three patients suffered intracerebral hemorrhage.

Bleeding episodes were classified according to GUSTO score; 13 (20%) were minor, 38 (58%) moderate and 15 (23%) severe. The first day of bleeding was day 1 or 2 in 50 (76%) out of the 66 patients, who experienced bleeding. Of these 50 bleedings, 42 (84%) were categorized as moderate or severe.

Of the patients bleeding later than day 1 or 2, eight (12%) had their first bleeding event on day 4 (± 1), five (8%) on day 7 (± 1) and three (4%) patients bled after day 8.

Bleedings were equally distributed among ECMO indication types as 19/31 (61%) VV-ECMO, 20/30 (67%) VA-ECMO and 27/39 (69%) ECPR patients experienced at least one minor, moderate or severe bleeding (p = 0.78). However, the overall prevalence of moderate and severe bleedings was higher among all VA-ECMO patients than among VV-ECMO patients (34/69, 49% vs 8/31, 26%, p = 0.03).

### Bleedings and coagulation parameters

Comparison of coagulation parameters between the 42 patients suffering moderate or severe bleeding on day 1 or 2 and the 58 patients with no or minor bleeds on day 1 or 2 are depicted in Table 3.

**Table 3:** Dynamic and standard coagulation parameters in patients during extracorporeal membrane oxygenation therapy divided according to severe or moderate bleeding on day 1 or 2 (n = 42) versus minor or no bleeding day 1 or 2 (n = 58). All data presented as median (interquartile range). Significant values are in bold.

	Bleeding n = 42	No bleeding n = 58	p-value
Multiplate*, AUC, all patients (78 healthy controls)			
ADP (870 [740–998] AU*min)	159 (86–336)	367 (170–584)	**< 0.001**
ASPI (1069 [961–1178] AU*min)	162 (96–399)	438 (180–605)	**< 0.001**
TRAP (1330 [1210–1463] AU*min)	530 (247–813)	698 (465–1046)	**0.01**
Multiplate*, AUC, Statistical analyses performed without patients with platelet count < 100 × 10^9^/L and treated with platelet inhibitors			
ADP (807 [740–998] AU*min)	216 (143–378) n = 14	449 (365–656) n = 31	**< 0.001**
ASPI (1069 [961–1178] AU*min)	373 (167–443) n = 15	576 (452–819) n = 29	**< 0.001**
TRAP (1330 [1210–1463] AU*min)	640 (355–889) n = 19	803 (541–1099) n = 29	0.10
ROTEM EXTEM* (reference interval)			
CT (38–79 s)	82 (70–99)	85 (74–97)	0.64
MAXVEL (2–22 mm/s)	10 (8–13)	16 (10–19)	**< 0.001**
t-MAXVEL (48–154 s)	140 (112–171)	124 (104–165)	0.36
MCF (50–72 mm)	56 (51–62)	63 (57–69)	**< 0.001**
ROTEM INTEM*			
CT (100–240 s)	225 (202–256)	203 (172–232)	**0.02**
MAXVEL (11–25 mm/s)	11 (8–14)	14 (11–19)	**< 0.01**
t-MAXVEL (147–223 s)	258 (228–295)	232 (205–268)	**0.04**
MCF (50–72 mm)	57 (51–62)	63 (56–67)	**< 0.01**
ROTEM FIBTEM* MCF (9–25 mm)	12 (8–18)	18 (11–25)	**0.047**
ROTEM HEPTEM* CT, s	202 (173–237)	191 (172–227)	0.30
ROTEM platelet MCE	112 (91–138)	155 (117–192)	**< 0.01**
Coagulation and thrombin generation parameters			
Platelet count (145–400 × 10^9^/L)	134 (69–174)	152 (104–210)	**0.04**
INR (< 1.2)	1.5 (1.3–1.7)	1.4 (1.2–2.1	0.59
Antithrombin (80–120%)	60 (48–78)	70 (60–82)	**0.02**
Fibrinogen (187–408 mg/dL)	221 (160–340)	306 (241–449)	**< 0.01**
APTT (20–29 s)	50 (33–68)	40 (33–50)	0.07
D-dimer (< 0.80 mg/L FEU)	10.7 (4.3–19)	7.2 (3.1–17.1)	0.25
TAT (≤ 13 µg/L)	19.7 (9.9–53.2)	15.8 (9.5–32.9)	0.34
Transfusion, day 1, mL			
Red blood cells	600 (282–1536)	0 (0–300)	**< 0.0001**
Fresh frozen plasma	0 (0–900)	0 (0–0)	**< 0.01**
Platelet concentrate	0 (0–440)	0 (0–0)	**< 0.001**

*ROTEM^®^ and Multiplate analysis was performed in 36 bleeding and 51 non-bleeding patients.

*AUC* area under the curve, *ADP* adenosine diphosphate, *ASPI* arachidonic acid induced aggregation, *TRAP* thrombin-receptor-agonist-peptide-6, *CT* clotting time, *MCF* maximum clot firmness, *MAXVEL* maximum velocity, *t-MAXVEL* time to MAXVEL, *MCE* maximum clot elasticity, *INR* international normalized ratio, *APTT* activated partial thrombin time, *TAT* thrombin–antithrombin complex.

Bleeding patients had lower platelet aggregation than patients without bleeding (all p-values ≤ 0.001). When excluding patients treated with platelet inhibitors and/or with platelet count < 100 × 10^9^/L, a significant difference was preserved for ADPtest and ASPItest (both p < 0.001). Consistent with this, platelet MCE was lower in bleeding patients than in non-bleeding patients (p < 0.01), as was platelet count (p = 0.04).

ROTEM^®^ measurements revealed lower EXTEM MCF (p < 0.001) and MaxVel (p < 0.001) in patients with moderate to severe bleeding on day 1 or 2 than in non-bleeding patients on day 1 or 2. All INTEM parameters were more hypocoagulable in bleeding patients than in non-bleeding patients (CT p = 0.02, MAXVEL p < 0.01, t-MAXVEL p = 0.04). However, the prevalence of bleeding among patients with INTEM CT above reference range was the same as in patients with INTEM CT within reference range (54% vs 37%, p = 0.15, Odds ratio (OR) 2.1 (95% CI 0.8–5.6)). Likewise, APTT measurements > 65 s were not associated with bleeding (63% vs 38%, p = 0.07, OR 1.8 (95% CI 0.7–4.8)).

Plasma levels of fibrinogen were lower in bleeding patients than in non-bleeding patients (p < 0.01) as was FIBTEM MCF (p = 0.05). However, looking at the lower reference level, the prevalence of patients with fibrinogen < 187 mg/dL was higher in bleeding patients than in non-bleeding patients (43% vs 9%, p < 0.001, OR 7.7 (95% CI 2.6–20.2)), while this was not the case for patients with FIBTEM MCF < 9 mm (25% vs 12%, p = 0.15, OR 2.5 (95% CI 0.8–8.2)).

EXTEM CT and INR did not differ between bleeding and non-bleeding patients (p = 0.74 and p = 0.59), indicating preserved clot initiation. Likewise, TAT levels were comparable between bleeding and non-bleeding patients (p = 0.34).

Patients with moderate or severe bleeding on day 1 or 2 (n = 42) received more blood products on day 1 than patients not bleeding (n = 58) (red blood cells p < 0.001, fresh frozen plasma p < 0.01, platelet concentrate p = 0.001) (Table 3). Few patients received hemostatic medications. During day 1 and 2, one patient received prothrombin complex concentrate (Octaplex^®^), three received fibrinogen concentrate and two patients received vitamin K. None received recombinant activated coagulation factor VII (NovoSeven^®^).

### Thrombotic complications

Fifteen patients were diagnosed with either thrombotic complications (n = 7), clots in the oxygenator (n = 7) or both (n = 1). Seven of the eight patients with thrombotic complications had signs of microthrombosis (critical peripheral ischemia), while one of these patients also had both vena cava inferior thrombosis as well as arterial thrombosis. Five of eight patients had thrombosis detected within the first 2 days, possibly related to infection and sepsis. In four patients, resection or amputation of thrombotic tissue was either planned or performed.

Oxygenator clot was observed 10 times in eight patients during the study period. In four patients, the oxygenator clot resulted in oxygenator change, and in one of these patients, the oxygenator was changed twice.

### Mortality

Of all patients, 48% died, either during ECMO treatment (n = 33) or within 30 days after successful weaning (n = 15); ECPR patients had the highest mortality of 67% (Table 1). In 33% of all patients, ECMO treatment was terminated due to death. Among the patients bleeding on day 1 or 2, more patients died (21/42) than patients not bleeding day 1 or 2 (12/58) (50% vs 21%, p = 0.003, OR 3.8 (95% CI 1.6–8.4)). When counting days of bleeding from day 1 to 7, patients that died had more days of bleeding than patients alive 30 days after weaning (median 2 (IQR 0–4) vs 0 (0–2). A total of 23 patients died within the first 7 days. Of these, 10 patients had a bleeding event on their last day alive. The direct cause of death was not registered. Thrombosis or oxygenator clot (in total 15 patients) was not associated with death (5/15, 33% vs 42/85, 51%, p = 0.27).

## Discussion

The present study reveals a multi-faceted coagulopathy in bleeding ECMO patients. Primarily, platelet aggregation is reduced, but also clot propagation and fibrinogen levels are lower in bleeding patients than in non-bleeding patients. Bleeding episodes occurred early on during the course of ECMO therapy and these early bleeding episodes were associated with mortality.

The present study provides new essential knowledge of importance for the clinical management as it demonstrates low platelet aggregation to be associated with moderate to severe bleeding on day 1 and 2 after initiation of ECMO therapy, irrespectively of platelet count above or below 100 × 10^9^/L. Patients with platelet count < 100 × 10^9^/L showed a higher prevalence of bleeding, pointing towards platelet count as well as platelet aggregation being implicated in bleeding during ECMO therapy, particularly in the early phase of ECMO therapy. This association between platelet count < 100 × 10^9^/L, decreased platelet aggregation and the presence of bleeding stresses the need for more focus on platelets during ECMO therapy. Previous studies revealed low platelet count^17, 26^, acquired von Willebrand disease within the first day of therapy^27^, and decreased platelet aggregation during ECMO therapy^18, 27–29^. Tauber et al. found decreased platelet aggregation to be associated with a higher need for transfusion of red blood cells^28^. Transfusion of red blood cells may, however, depend on other factors than bleeding, e.g., hemolysis, specific transfusion limits, and relative ischemia. In another study, Siegel et al. evaluated 30 ECMO patients and found decreased platelet aggregation within 3 days of treatment to be associated with death, but associations with bleeding were not presented and patients receiving platelet inhibitors were included in the analysis. The data in the present study were analyzed with and without patients receiving platelet inhibitors.

With regard to global hemostasis ECMO patients, all ROTEM^®^ parameters changed in a hypocoagulable direction, except for clot firmness in the FIBTEM assay. Bleeding patients had slower clot propagation and lower clot firmness than non-bleeding patients, but clot firmness results remained within the reference range. A larger proportion of bleeding patients than non-bleeding patients had plasma fibrinogen levels below reference range. This is in accordance with a smaller study by Laine et al.^15^, where lower plasma fibrinogen was reported in severely bleeding patients than in non-bleeding patients, although not significantly. Hence, the present study strengthens the indication of low plasma fibrinogen level as an additional risk factor for bleeding in ECMO patients and challenges previous levels of substitution suggested by others, going as low as 150 mg/dL or FIBTEM MCF < 8 mm^16^. Overall, congruity was shown between standard coagulation tests and ROTEM^®^ measurements. However, plasma fibrinogen was stronger associated with bleeding than FIBTEM clot firmness and, confirming previous studies^13, 30^, the correlation between all APTT and INTEM CT measurements was only moderate. Hence, the present study does not support ROTEM® as a routine part of coagulation monitoring during ECMO therapy, but strengthens the indication for measuring plasma fibrinogen along with standard coagulation parameters (INR, APTT, platelet count) when monitoring hemostasis in ECMO patients.

The prevalence of moderate and severe bleeding episodes in the present study was comparable to other studies as summarized by Jiritano et al.^26^. The present study also confirms that bleeding is an early event during ECMO treatment and the risk of bleeding after day 3 is low, as found by others^31^. Of importance, bleeding during ECMO is associated with mortality, which is demonstrated by the present and other studies^5, 6^.

The present study only detected symptomatic thrombotic events. Thrombosis or oxygenator clot were registered in 15% of the patients, which is somewhat lower than found in register studies^5, 6^. Generally, thrombotic events in ECMO patients are possibly highly underestimated by clinical evaluation^32^.

The strengths of the present study comprise a large cohort compared with previous studies on bleeding and thrombosis during ECMO therapy and with similar patient characteristics in particular concerning age, predominantly male sex, comorbidities, duration, and indications for ECMO therapy^4–6^. Patients in the present study were extensively examined with conventional and dynamic coagulation assays evaluating primary and secondary hemostasis including in vivo thrombin generation. Evaluation of platelet aggregation measures were performed with and without exclusion of patients receiving antiplatelet medicine and platelet count above and below 100 × 10^9^/L, as platelet aggregation is negatively affected by platelet count < 100 × 10^9^/L^33^ Further, patients were followed during their entire ECMO run and until 30 days after weaning. Based on detailed clinical information the association with bleeding and thrombosis could be investigated.

This study also has limitations. A cause-and-effect relationship could not be established as coagulation measurements and bleeding events were concurrent. Registration of bleeding and thrombosis relied upon medical charts leading to possible underreporting. The registration and grading of bleedings were however, supported by systematic registrations of transfusion requirements and hemodynamic data. The present study did not perform ultrasound following decannulation. Hence, incidental deep venous thrombosis at cannulation sites were not found and these constitute a large part of thrombotic events in previous reports^32^. Due to high early mortality causing drop-out and heterogeneity in the course of disease, repeated measurements analysis was not feasible.

In conclusion, low fibrinogen levels and decreased platelet aggregation, irrespective of platelet count, were associated with bleeding on day 1–2 after ECMO initiation. Congruity existed between ROTEM® measurements and standard coagulation assays, but plasma fibrinogen measurements had a stronger association with bleeding than ROTEM^®^ measurements and the correlation between APTT and INTEM CT measurements was only moderate. The present study does not support ROTEM^®^ analysis as a routine part of coagulation monitoring during ECMO therapy.

## Data Availability

The data that support the findings of this study are available from the corresponding author C.L.H but restrictions apply to the availability of these data, which were used under license for the current study, and so are not publicly available. Data are however available from the authors upon reasonable request to the corresponding author C.L.H and with permission of Department of Intensive Care, Aarhus University Hospital.
